# Association of conventional cigarette smoking, heated tobacco product use, and dual use with hearing loss: A working population-based study

**DOI:** 10.18332/tid/187302

**Published:** 2024-05-23

**Authors:** Huan Hu, Haruka Miyake, Takeshi Kochi, Toshiaki Miyamoto, Hiroko Okazaki, Isamu Kabe, Aki Tomizawa, Tohru Nakagawa, Toru Honda, Shuichiro Yamamoto, Maki Konishi, Shohei Yamamoto, Yosuke Inoue, Seitaro Dohi, Tetsuya Mizoue

**Affiliations:** 1 Research Center for Prevention from Radiation Hazards of Workers, National Institute of Occupational Safety and Health, Kanagawa, Japan; 2 Department of Epidemiology and Prevention, Center for Clinical Sciences, National Center for Global Health and Medicine, Tokyo, Japan; 3 Furukawa Electric Co., Ltd., Tokyo, Japan; 4 East Nippon Works Kimitsu Area, Nippon Steel Corporation, Chiba, Japan; 5 Mitsui Chemicals, Inc., Tokyo, Japan; 6 KUBOTA Corporation Co., Ltd., Ibaraki, Japan; 7 Health Design Inc., Tokyo, Japan; 8 Hitachi Health Care Center, Hitachi, Ltd., Ibaraki, Japan

**Keywords:** heated tobacco product, cigarette, hearing loss

## Abstract

**INTRODUCTION:**

Although conventional cigarette smoking has been linked to an increased risk of hearing loss, the association between heated tobacco products (HTPs) and hearing loss is unknown. The objective of this study was to investigate the association between cigarette and HTP use and hearing loss.

**METHODS:**

This cross-sectional study examined the data of 7769 employees from five companies (Study I) and 34404 employees from a large company (Study II), all participants in the Japan Epidemiology Collaboration on Occupational Health Study. The participants were categorized into five groups based on their self-reported tobacco use: never smokers, former smokers, exclusive cigarette smokers, exclusive users of HTPs, and those who used both cigarettes and HTPs. Hearing levels were measured using pure-tone audiometry at 1 and 4 kHz frequencies. Separate analyses were carried out for each study, and the results were then combined using fixed-effect models to pool the estimates.

**RESULTS:**

The analysis included 42173 participants, with a prevalence of 12.9% for exclusive cigarette smoking, 9.8% for exclusive HTP use, and 5.5% for dual use. The pooled adjusted odds ratios with 95% confidence intervals for unilateral hearing loss at 4 kHz were 1.21 (95% CI: 1.10–1.33) for former smokers, 1.83 (95% CI: 1.64–2.05) for exclusive cigarette smokers,1.46 (95% CI: 1.28–1.67) for exclusive HTP users, and 1.66 (95% CI: 1.41–1.96) for dual users, compared to never smokers. Additionally, the adjusted odds ratios for hearing loss at 4 kHz among exclusive cigarette smokers, exclusive HTP users, and dual users increased with the intensity of cigarette/HTP consumption (all p for trend <0.001). No significant associations were found between exclusive HTP use, dual use, and hearing loss at 1 kHz, apart from exclusive cigarette smoking.

**CONCLUSIONS:**

In this cross-sectional study, associations were found between exclusive cigarette smoking, exclusive HTP use, dual use, and hearing loss, particularly at 4 kHz. Further research is needed to confirm these findings.

## INTRODUCTION

New tobacco-related products, such as heated tobacco products (HTPs) and e-cigarettes, are gaining popularity worldwide^1,2^. Japan has emerged as one of the largest markets for HTPs, where nearly half of current tobacco product users reported using HTPs exclusively or concurrently with other tobacco products^3^. HTPs heat tobacco within a specific temperature range and emit harmful or potentially harmful compounds similar to those found in cigarette smoke, albeit at lower concentrations^4^. However, the health effects of these products remain largely unknown.

Hearing loss, which affected an estimated 1.57 billion people globally in 2019^5^, is a major public health challenge, and has been associated with conventional cigarette smoking^6-8^. Ototoxic chemicals in cigarette smoke such as nicotine, toluene, and benzene are also found in HTP aerosols^4,9^, raising concerns about the adverse effects of HTPs on hearing. In addition, the effect of dual use on hearing requires attention, given that approximately half of HTP users continue to smoke cigarettes^3^. To the best of our knowledge, no previous studies have examined the association between HTP use and hearing loss. Therefore, we assessed the cross-sectional association between cigarette smoking, HTP use, dual use of cigarettes and HTPs, and hearing loss using data from a large working population in Japan.

## METHODS

This cross-sectional study used data from the ongoing Japan Epidemiology Collaboration on Occupational Health (J-ECOH) Study, involving multiple companies in Japan^6,10^. Participants underwent yearly health checkups, including self-administered questionnaires, physical exams (including hearing tests), and laboratory tests as mandated by the Industrial Safety and Health Act. Supplementary file Table 1 shows that, during Phase 3 of the J-ECOH Study (April 2018 to March 2021), five participating companies carried out an extra questionnaire survey targeting the use of tobacco-related products (Study I). Additionally, data on the use of these products were also collected from the 2019 health checkup conducted in another large company participating in the J-ECOH Study (Study II).

Prior to data collection, the J-ECOH Study was announced in each participating company using posters. Participants did not provide written or verbal informed consent; however, they were allowed to refuse or withdraw their participation at any time using an opt-out methodology. The study protocol, including the consent procedure, was approved by the Ethics Committee of the National Center for Global Health and Medicine, Japan (NCGM-G-001140).

### Participants

In Study I, 12846 workers, representing over 70% of the target population, completed the Phase 3 questionnaire, with 12672 also having health checkup data from the same fiscal year. Exclusions were made for missing smoking data, using e-cigarettes, missing hearing data, and missing covariates. In one company, the questionnaire survey was conducted more than six months after the health checkup in that fiscal year. Thus, we further excluded 84 people who reported having quit cigarette smoking in the past year to reduce potential misclassifications. In Study II, 49333 workers attended health checkups in 2019. We excluded participants with missing data on smoking, hearing, or covariates. Finally, 7769 participants from Study I and 34404 participants from Study II were included in the analyses, as shown in Supplementary file Figure 1.

### Outcome

As part of the annual health checkup, hearing tests were conducted in a quiet room by trained audiometric technicians using a pure-tone audiometer equipped with supra-aural headphones. Workers were screened for hearing thresholds exceeding 30 dB at 1 kHz and 40 dB at 4 kHz in each ear. In Japan, national health checkup guidelines mandate hearing tests at 1 kHz and 4 kHz, and a hearing threshold exceeding 30 dB at 1 kHz or 40 dB at 4 kHz is considered indicative of hearing impairment^11^. Consequently, in the Japanese working population, hearing is typically assessed only at these two frequencies (1 kHz and 4 kHz) to determine whether the thresholds exceed these specified levels.

In this study, hearing loss was defined separately for 1 kHz and 4 kHz. Consistent with previous studies from Japan^6,7,10,12^, hearing loss at 1 kHz was defined as a hearing threshold >30 dB in the worse-hearing ear, indicating unilateral hearing loss. For hearing loss at 4 kHz, it was defined as a hearing threshold >40 dB in the worse-hearing ear. Cases at 1 and 4 kHz groups were not mutually exclusive. Alternatively, we defined hearing loss in both ears as bilateral hearing loss.

### Main exposure

Within the J-ECOH Study, both Study I and Study II collected data on the use of new tobacco-related products among employees (Supplementary file Table 1). Study I implemented a specialized questionnaire in five participating companies to assess the usage of these products. In contrast, Study II focused on a large company within the J-ECOH Study, using a questionnaire developed by the company itself.

In Study I, we assessed cigarette smoking through the question: ‘Do you smoke conventional cigarettes?’. Participants were provided with the following response options: ‘never’, ‘quit ≥5 years ago’, ‘quit < 5 years ago’, and ‘smoke 1–5’, ‘smoke 6–10’, ‘smoke 11–20’, or ‘smoke >20’ cigarettes per day. To identify new tobacco-related product use, we asked participants: ‘Did you use new tobacco-related products in the last month?’. They were instructed to select one or more of the following responses: ‘no’, ‘used HTPs (e.g. IQOS, Ploom Tech, glo)’, and ‘used e-cigarettes’. In Japan, the sale of nicotine-containing e-cigarettes is prohibited under the Pharmaceutical Affairs Law, and the non-nicotine e-cigarettes available in the market are not classified as tobacco products. Consequently, we excluded e-cigarette users (n=116) from our analysis. The remaining participants were divided into five groups: never smokers, former smokers, exclusive HTP users, dual users of cigarettes and HTPs, and exclusive cigarette smokers, as in previous publications^13,14^.

In Study II, a slightly different questionnaire was used. Participants were asked about their current tobacco product usage, including conventional cigarettes, e-cigarettes, and HTPs, with response options of: ‘never’, ‘past use’, or ‘current use’. For current users, a follow-up question identified their specific product choices: ‘conventional cigarettes only’, ‘New tobacco-related products only (e-cigarettes and/ or HTPs)’, or ‘both conventional cigarettes and new tobacco-related products’. The questionnaire did not allow for the differentiation between HTPs and e-cigarettes based on the participants’ responses. Given the limited use of e-cigarettes in Japan (about 1%) and their non-classification as tobacco products in the country^15,16^, smoking status in Study II was categorized following the same approach as Study I, presuming that users of new tobacco-related products were predominantly HTP users. Study II also asked the number of cigarettes/HTPs used per day, further categorizing exclusive HTP users, dual users, and exclusive cigarette smokers into three groups based on daily consumption (1–10, 11–20, ≥21) to examine the dose-response relationship with hearing loss.

### Covariates

Covariates were chosen based on factors suggested to be associated with hearing loss. For our analysis, we considered a range of covariates, including age, sex, body mass index (BMI, kg/m^2^)^10,17^, leisure-time physical activity^17^, alcohol consumption^18^, hypertension^19,20^, diabetes^20,21^, and occupational noise exposure^22^. Participants had their height measured and their body weight using a scale while dressed in light clothing and without shoes. Alcohol consumption was categorized into four groups: non-drinkers, those who consumed: <1 *go*, 1 to <2 *go*, or ≥2 *go* per day, where 1 *go* of sake contains approximately 23 g of ethanol. Leisure-time physical activity varied, and in Study I was categorized as: 0, >0 to <3, 3 to <10, or ≥10 MET-hours per week; in Study II it was classified as either ≥150 minutes per week, or <150 minutes per week. Exposure to occupational noise was determined by a self-administered questionnaire, with participants indicating either ‘yes’ or ‘no’. In Study I, occupational noise exposure was identified based on participants reporting specific health checkups for workplace noise hazards in the past three years, while in Study II, it was identified if participants reported that their workplace was noisy. Hypertension was defined as systolic blood pressure ≥140 mmHg, diastolic blood pressure ≥90 mmHg, or receiving medical treatment for hypertension^23^. Diabetes was defined as fasting blood glucose ≥126 mg/dL, HbA1c level ≥6.5%, or receiving medical treatment for diabetes^24^.

### Statistical analyses

Descriptions of the study participants include mean values and standard deviations for continuous variables, and percentages for categorical ones. Logistic regression analysis was employed to calculate adjusted odds ratios (AORs) and 95% confidence intervals (CIs) to assess the association between the use of HTPs and hearing loss. The initial model accounted for variables such as age and sex. We further adjusted for known and potentially important factors of hearing loss, such as alcohol intake, physical activity during leisure, exposure to noise at work, BMI, diabetes, and hypertension, to mitigate confounding bias. Separate analyses were performed for Studies I and II, followed by a combined analysis using a fixed-effects model due to the homogeneity in study designs and methods of data collection. The choice of a fixed-effects model was based on the fact that both studies included participants from the J-ECOH Study, sharing similar characteristics, which ensured consistency in our analysis. Moreover, a dose-response assessment was conducted using the data from Study II.

The analysis of individual-level data was carried out using SAS version 9.4 (SAS Institute, Cary, NC, USA), and a meta-analysis was performed utilizing Stata version 14 (StataCorp, College Station, TX, USA). All statistical tests were two-sided, with significance set at a p<0.05.

## RESULTS

A total of 42173 participants with a mean age of 47.0 ± 10.6 years (84.0% men) were included in the analysis. The prevalence rates of exclusive cigarette smoking, exclusive HTP use, and combined use were 12.9 %, 9.8 %, and 5.5%, respectively. The prevalence rates of unilateral hearing loss at 1 kHz and 4 kHz were 3.8% and 8.3%, respectively. Table 1 shows that the participants in Studies I and II had similar characteristics. Participants who used only HTPs were younger than those who smoked cigarettes exclusively. Compared with never smokers, exclusive HTP users were more likely to be male and have a worse risk factor profile for hearing loss, such as BMI, diabetes, and occupational noise exposure.

**Table 1:** Participant characteristics in a cross-sectional study of Japanese workers, 2018–2020 (N=42173)

*Characteristics*	*Never smoker %*	*Past smoker %*	*Current use of tobacco-related products*		
			*Exclusive cigarette smoker %*	*Exclusive HTP user %*	*Dual user %*
**Study I**					
Total, n	3657	2178	925	444	565
Age (years), mean ± SD	44.1 ± 11.6	51.0 ± 8.8	47.5 ± 10.5	44.5 ± 9.8	43.7 ± 10.9
Male	77.8	96.8	96.4	98.4	98.4
Body mass index (kg/m^2^), mean ± SD	23.5 ± 3.9	24.3 ± 3.3	24.1 ± 3.7	24.5 ± 3.7	24.7 ± 4.0
Alcohol consumption	70.0	82.2	77.8	76.1	80.2
Leisure-time physical activity (≥10 METs-hours/week)	27.8	32.1	21.8	24.3	22.8
Hypertension	20.8	35.5	28.5	25.9	25.8
Diabetes	6.2	11.3	10.5	8.6	13.5
Occupational noise exposure	5.6	10.1	10.9	13.5	11.7
**Study II**					
Total, n	15987	8422	4513	3710	1772
Age (years), mean ± SD	45.1 ± 11.4	50.6 ± 9.0	48.6 ± 9.3	46.1 ± 8.5	45.9 ± 9.7
Male	70.6	93.3	94.1	94.6	94.8
Body mass index (kg/m^2^), mean ± SD	23.6 ± 4.0	24.2 ± 3.5	24.0 ± 3.8	24.4 ± 3.9	24.6 ± 4.0
Alcohol consumption	52.2	74.7	67.5	69.1	69.0
Leisure time physical activity (≥150 minutes/week)	14.2	19.6	12.8	15.9	12.8
Hypertension	17.9	29.5	23.5	20.0	22.1
Diabetes	6.9	11.5	12.2	10.5	12.0
Occupational noise exposure	15.1	18.4	21.4	20.0	24.2

**Figure 1:**
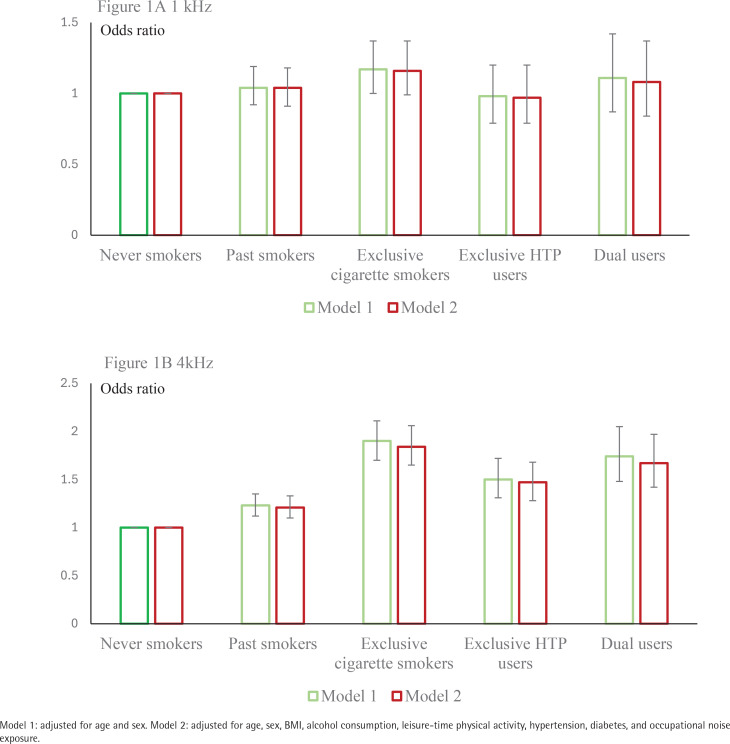
Pooled adjusted odds ratios (95% confidence intervals) for unilateral hearing loss among Japanese workers, 2018–2021 (N=42173)

Figure 1 displays significant associations between exclusive cigarette smoking, exclusive HTP use, dual use of cigarettes and HTPs, and unilateral hearing loss at 4 kHz. In contrast, no significant associations were observed for hearing loss at 1 kHz, apart from exclusive cigarette smoking. Further, exclusive HTP use appeared to have a weaker association with hearing loss at 4 kHz than exclusive cigarette smoking. The magnitude of associations of dual-use and exclusive cigarette smoking with hearing loss at 4 kHz was similar. As shown in Supplementary file Table 2, compared with never smokers, the AORs (95% CIs) for hearing loss at 4 kHz among former smokers, exclusive cigarette smokers, exclusive HTP users, and dual users were 1.35 (95% CI: 1.08–1.69), 2.01(95% CI: 1.52–2.65), 1.62 (95% CI: 1.06–2.47), and 1.96 (95% CI: 1.36–2.81) in Study I, as well as 1.17 (95% CI: 1.06–1.31), 1.80 (95% CI: 1.60–2.03), 1.44 (95% CI: 1.25–1.67), and 1.59 (95% CI: 1.33–1.91) in Study II, respectively. The pooled AORs (95% CIs) were 1.21 (95% CI: 1.10–1.33), 1.83 (95% CI: 1.64–2.05), 1.46 (95% CI: 1.28–1.67), and 1.66 (95% CI: 1.41–1.96), respectively. Analysis showed no evidence of statistical heterogeneity, with I^2^=0% and p=0.62, indicating consistent results across studies.

**Figure 2:**
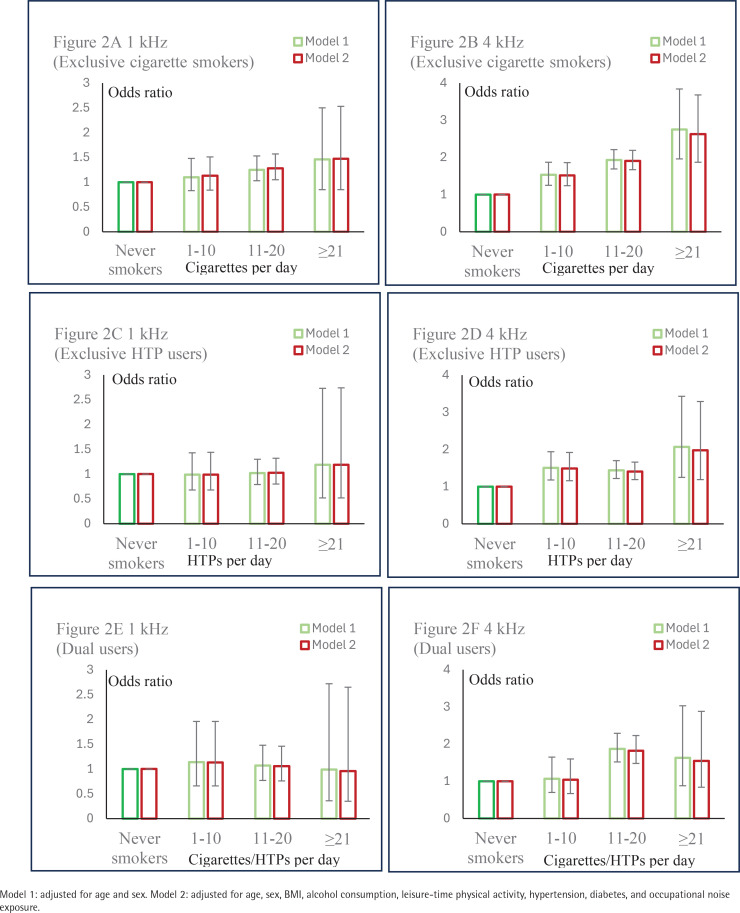
Dose-response relationship between tobacco product use and unilateral hearing loss in Japanese workers, 2018–2021 (Study II, N=25926)

Figure 2 further shows that the AORs for unilateral hearing loss at 4 kHz increased by intensity of cigarette/HTP use in exclusive cigarette smokers, exclusive HTP users, and dual users. As shown in Supplementary file Table 3, among exclusive cigarette smokers, the AORs (95% CIs) for people who smoked 1–10, 11–20, and >20 cigarettes per day were 1.52 (95% CI: 1.24–1.86), 1.91 (95% CI: 1.67–2.19), and 2.63 (95% CI: 1.87–3.68), respectively (p for trend <0.001). Among exclusive HTP users, the AORs (95% CIs) for people who used 1–10, 11–20, and >20 HTPs per day were 1.49 (95% CI: 1.16–1.92), 1.41 (95% CI: 1.19–1.66), and 1.98 (95% CI: 1.19–3.29), respectively (p for trend <0.001). Among dual users, the AORs (95% CIs) for people who used 1–10, 11–20, and >20 cigarettes/HTPs per day were 1.04 (95% CI: 0.67–1.60), 1.82 (95% CI: 1.48–2.23), and 1.55 (95% CI: 0.84–2.88), respectively (p for trend <0.001).

When defining hearing loss in both ears, we found that exclusive cigarette smoking, exclusive HTP use, and dual-use were significantly associated with bilateral hearing loss at 4 kHz (Supplementary file Tables 4 and 5). However, we did not observe a clear difference in the strength of the association between exclusive HTP users, dual users, and exclusive cigarette smokers, possibly due to the small number of cases with bilateral hearing loss.

## DISCUSSION

This study analyzed a large working population and found that exclusive cigarette smoking, exclusive HTP use, and dual-use were associated with increased odds of hearing loss in a dose-response manner at 4 kHz. Moreover, the association between exclusive HTP use and hearing loss at 4 kHz was weaker than between exclusive cigarette smoking and hearing loss. No significant association with hearing loss was found at 1 kHz, except for exclusive cigarette smoking. This study provides the first test of whether there are associations of HTP use with hearing loss.

The finding of a significant dose-response association between HTP use and hearing loss at 4 kHz is in line with previous research demonstrating the negative impact of cigarette smoking on hearing^6-8^. For example, in a cohort study of male office workers in Japan, cigarette smoking was associated with a higher risk of hearing loss at 4 kHz in a dose-response manner in terms of pack-years^7^. Our study showed that exclusive HTP users had increased odds of hearing loss at 4 kHz and that the odds increased with the number of HTPs used per day. These findings suggested that HTP use may have adverse effects on hearing.

There are several potential mechanisms by which HTP leads to hearing loss. First, HTPs deliver nicotine to aerosols at levels comparable to those in cigarettes^9^. Nicotine has a direct ototoxic effect on the outer hair cells responsible for high-frequency hearing^25^. Second, HTP aerosols also contain other ototoxic chemicals, such as toluene and benzene, which can harm hair cells in the inner ear^4,9,26^. Additionally, HTPs can increase oxidative stress, which causes damage to the outer hair cells, resulting in hearing loss^27,28^. HTP aerosols have nicotine levels comparable to cigarette smoke but lower levels of other ototoxic chemicals and oxidative toxicity^4,9,27^, which might partially account for the weaker association between exclusive HTP use and hearing loss in comparison to exclusive cigarette smoking.

It is unclear whether the concurrent use of HTPs and cigarettes can reduce smoking-related harm, despite the common belief among tobacco users that HTPs are less harmful than cigarettes among tobacco users^29^. In the present study, the magnitude of the association between dual use of cigarettes and HTPs and hearing loss at 4 kHz was comparable to that of exclusive cigarette smoking. One possible explanation is that dual users do not smoke cigarettes less than exclusive cigarette smokers. In Study I, where data on cigarette consumption were available, the proportions of participants who smoked 1–10, 11–20, and ≥21 cigarettes per day did not differ significantly between dual users (36.4%, 56.3%, and 7.3%, respectively) and exclusive cigarette smokers (35.1%, 56.2%, and 8.7%, respectively) (p=0.23). These observations suggest that the concurrent use of cigarettes and HTPs may not substantially lead to decreased cigarette consumption and, thus, it may not significantly reduce the risk of hearing loss.

We did not observe any significant association between HTP use and hearing loss at 1 kHz; however, previous studies have shown that cigarette smoking is associated with low-frequency hearing loss^6,7^. The reason for this difference is unclear, but the ototoxic effects of HTPs may be less potent than those of conventional cigarettes, as previously discussed. It is also important to note that the analysis may have been underpowered because of the relatively small number of cases of hearing loss at 1 kHz, particularly among exclusive HTP users and dual users. Additional research is required to investigate the association between HTP use and low-frequency hearing loss.

### Strengths and limitations

Our study has several strengths, including a well-powered comparison of never, past, and current smokers, the use of audiometry to measure hearing thresholds, and comprehensive covariate adjustment. However, our study also has several limitations. First, the study’s cross-sectional design does not allow for the establishment of causality between HTP use and hearing loss. Second, smoking habits were self-reported and not biologically verified (e.g. exhaled carbon monoxide or salivary cotinine), which may lead to misclassification. Third, hearing tests were limited to 1 and 4 kHz frequencies during health checkups, which may have missed hearing loss at other frequencies. Fourth, residual and unmeasured confounding factors, such as non-occupational noise exposure, may have affected our results. Fifth, due to the absence of uniformly collected data on otologic diseases, head injuries, or drugs that could affect hearing acuity, we could not exclude hearing loss cases potentially caused by these factors. Lastly, our study was conducted in the Japanese working population, so generalizations about other populations should be made cautiously.

## CONCLUSIONS

Our study found that exclusive cigarette smoking, exclusive HTP use, and dual-use were associated with hearing loss, especially at 4 kHz. While our results highlight a potential link between HTP use and hearing loss, future prospective cohort studies are needed to confirm this relationship.

## Supplementary Material



## Data Availability

The data supporting this research are available from the authors on reasonable request.
