# Supplementary material for: Association of conventional cigarette smoking, heated tobacco product use, and dual use with hearing loss: A working population-based study
Source: Tob Induc Dis. 2024 May 23;22:10.18332/tid/187302. doi: 10.18332/tid/187302 (PMC11113009; doi:10.18332/tid/187302)
Supplement: Supplementary file 1 [file TID-22-85-s1.pdf]

Supplementary file Figure 1 Flowchart of a cross-sectional study of Japanese workers, 2018-2020 (N=42173)

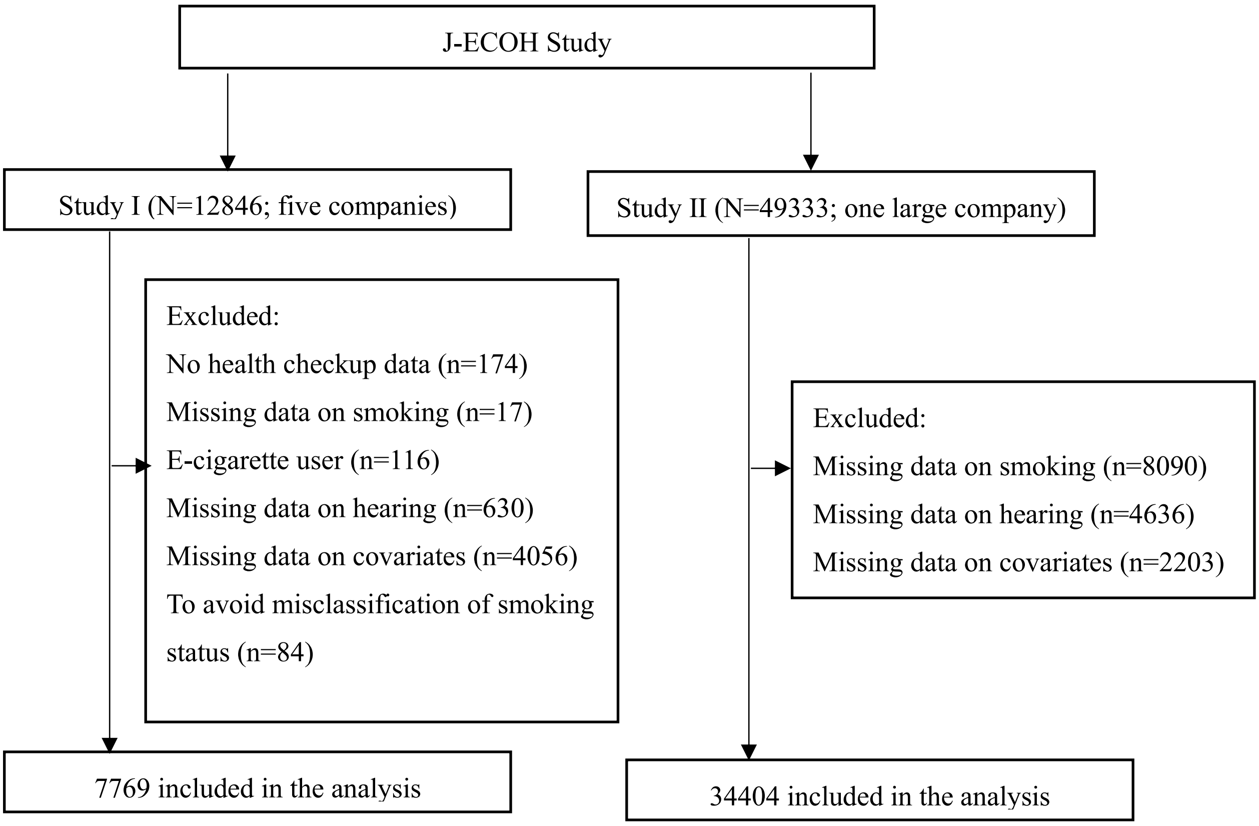

Supplementary file Table 1 The J-ECOH Study flowchart

	Phase 1	Phase 2	Phase 3	Phase 4
	2012-14	2015-17	2018-20	2021-23
Annual health check-up	✓	✓	✓	✓
Registrations of cardiovascular disease, long-term sick leave, and death		✓	✓	✓
Health survey (selected companies)			✓	✓
The study initially encompassed more than 100,000 workers from 12 companies. Five and four companies participated in the health survey in Phase 3 and Phase 4, respectively.				

Supplementary file Table 2 Adjusted odds ratios (95% confidence intervals) for unilateral hearing loss among Japanese workers, 2018-2020 (N=42173)

	Current use of tobacco-related products					Heterogeneity[a] P, I^2 (%)
	Never smoker	Past smoker	Exclusive cigarette smoker	Exclusive HTP user	Dual user	
**Study I**						
1 kHz						
No. of cases /No. of people	105/3657	107/2178	35/925	5/444	19/563	
Model 1	Reference	1.13 (0.85-1.51)	1.07 (0.71-1.61)	0.40 (0.16-1.01)	1.26 (0.75-2.09)	
Model 2	Reference	1.06 (0.80-1.42)	1.01 (0.67-1.52)	0.38 (0.15-0.95)	1.15 (0.69-1.91)	
4 kHz						
No. of cases /No. of people	163/3657	249/2178	113/925	33/444	49/565	
Model 1	Reference	1.42 (1.14-1.76)	2.14(1.63-2.81)	1.75 (1.15-2.66)	2.10 (1.47-3.01)	
Model 2	Reference	1.35 (1.08-1.69)	2.01 (1.52-2.65)	1.62 (1.06-2.47)	1.96 (1.36-2.81)	
**Study II**						
1 kHz						
No. of cases /No. of people	544/15987	393/8422	209/4513	117/3710	61/1772	
Model 1	Reference	1.02 (0.89-1.18)	1.19 (1.01-1.42)	1.02 (0.83-1.26)	1.07 (0.81-1.41)	
Model 2	Reference	1.03 (0.89-1.19)	1.20 (1.01-1.42)	1.03 (0.83-1.27)	1.06 (0.80-1.39)	

4 kHz						
No. of cases /No. of people	895/15987	935/8422	596/4513	311/3710	172/1772	
Model 1	Reference	1.20 (1.08-1.33)	1.85 (1.65-2.08)	1.48 (1.28-1.70)	1.66 (1.39-1.99)	
Model 2	Reference	1.17 (1.06-1.31)	1.80 (1.60-2.03)	1.44 (1.25-1.67)	1.59 (1.33-1.91)	
**Pooled results**						
1 kHz						
Model 1	Reference	1.04 (0.92, 1.19)	1.17 (1.00, 1.37)	0.98 (0.79, 1.20)	1.11 (0.87, 1.42)	0.05, 73.4
Model 2	Reference	1.04 (0.91, 1.18)	1.16 (0.99, 1.37)	0.97 (0.79, 1.20)	1.08 (0.84, 1.37)	0.04, 77.0
4 kHz						
Model 1	Reference	1.23 (1.12, 1.35)	1.90 (1.70, 2.11)	1.50 (1.31, 1.72)	1.74 (1.48, 2.05)	0.46, 0.0
Model 2	Reference	1.21 (1.10, 1.33)	1.83 (1.64, 2.05)	1.46 (1.28, 1.67)	1.66 (1.41, 1.96)	0.62, 0.0

Model 1 adjusted for age and sex

Model 2 adjusted for age, sex, BMI, alcohol consumption, leisure-time physical activity, hypertension, diabetes, and occupational noise exposure

[a] For the exclusive HTP user group

Supplementary file Table 3 Dose-response relationship between tobacco product use and unilateral hearing loss in Japanese workers, 2018-2020 (Study II, N=25926)

	Never smoker	Number of cigarettes / HTPs used per day			P for trend*
		1-10	11-20	≥21	
**Exclusive cigarette smoking**					
1 kHz					
No. of cases / No. of people	544/15987	54/1372	139/2868	15/246	
Odds ratio (95% CI)					
Model 1	Reference	1.10 (0.83, 1.48)	1.25 (1.03, 1.53)	1.46 (0.85, 2.50)	0.01
Model 2	Reference	1.13 (0.84, 1.51)	1.28 (1.05, 1.57)	1.47 (0.85, 2.53)	0.008
4 kHz					
No. of cases / No. of people	895/15987	136/1372	405/2868	51/246	
Odds ratio (95% CI)					
Model 1	Reference	1.53 (1.25, 1.87)	1.93 (1.69, 2.21)	2.75 (1.96, 3.84)	<0.001
Model 2	Reference	1.52 (1.24, 1.86)	1.91 (1.67, 2.19)	2.63 (1.87, 3.68)	<0.001
**Exclusive HTP use**					

1 kHz					
No. of cases / No. of people	544/15987	31/1164	80/2385	6/141	
Odds ratio (95% CI)					
Model 1	Reference	0.99 (0.68, 1.43)	1.02 (0.79, 1.30)	1.19 (0.52, 2.73)	0.81
Model 2	Reference	0.99 (0.68, 1.44)	1.03 (0.80, 1.32)	1.19 (0.52, 2.74)	0.75
4 kHz					
No. of cases / No. of people	895/15,987	79/1,164	212/2,385	19/141	
Odds ratio (95% CI)					
Model 1	Reference	1.51 (1.18, 1.94)	1.44 (1.22, 1.70)	2.07 (1.25, 3.43)	<0.001
Model 2	Reference	1.49 (1.16, 1.92)	1.41 (1.19, 1.66)	1.98 (1.19, 3.29)	<0.001
**Dual use**					
1 kHz					
No. of cases / No. of people	544/15987	14/461	43/1201	4/101	
Odds ratio (95% CI)					
Model 1	Reference	1.14 (0.66, 1.96)	1.07 (0.77, 1.48)	0.99 (0.36, 2.72)	0.67
Model 2	Reference	1.13 (0.66, 1.96)	1.06 (0.76, 1.47)	0.96 (0.35, 2.65)	0.72

4 kHz					
No. of cases / No. of people	895/15987	24/461	135/1201	13/101	
Odds ratio (95% CI)					
Model 1	Reference	1.07 (0.70, 1.65)	1.87 (1.52, 2.29)	1.63 (0.88, 3.03)	<0.001
Model 2	Reference	1.04 (0.67, 1.60)	1.82 (1.48, 2.23)	1.55 (0.84, 2.88)	<0.001

Model 1 adjusted for age and sex

Model 2 adjusted for age, sex, BMI, alcohol consumption, leisure-time physical activity, hypertension, diabetes, and occupational noise exposure

*The P for trend was determined by modeling smoking intensity as a continuous variable, incorporating the median values of each group into the analysis.

Supplementary file Table 4 Adjusted odds ratios (95% confidence intervals) for bilateral hearing loss among Japanese workers, 2018-2020 (N=42173)

	Current use of tobacco-related products					Heterogeneity[a] P, I^2 (%)
	Never smoker	Past smoker	Exclusive cigarette smoker	Exclusive HTP user	Dual user	
**Study I**						
1 kHz						
No. of cases /No. of people	31/3657	38/2178	9/925	3/444	8/565	
Model 1	Reference	1.34 (0.81-2.21)	0.93 (0.43-2.05)	0.89 (0.26-2.97)	1.87 (0.83-4.20)	
Model 2	Reference	1.24 (0.75-2.04)	0.84 (0.38-1.87)	0.80 (0.24-2.67)	1.61 (0.71-3.63)	
4 kHz						
No. of cases /No. of people	73/3657	120/2178	56/925	15/444	23/565	
Model 1	Reference	1.30 (0.95-1.78)	2.11 (1.44-3.09)	1.83 (0.99-3.40)	2.18 (1.31-3.62)	
Model 2	Reference	1.28 (0.93-1.76)	2.02 (1.36-2.99)	1.72 (0.92-3.22)	2.06 (1.23-3.45)	
**Study II**						
1 kHz						
No. of cases /No. of people	184/15987	114/8422	67/4513	29/3710	14/1772	
Model 1	Reference	0.91 (0.71-1.18)	1.22 (0.91-1.64)	0.88 (0.58-1.31)	0.82 (0.47-1.43)	
Model 2	Reference	0.91 (0.70-1.18)	1.23 (0.91-1.66)	0.88 (0.58-1.32)	0.81 (0.46-1.41)	
4 kHz						

No. of cases /No. of people	330/15987	394/8422	229/4513	135/3710	77/1772	
Model 1	Reference	1.29 (1.10-1.51)	1.81 (1.51-2.16)	1.82 (1.47-2.25)	2.04 (1.57-2.66)	
Model 2	Reference	1.28 (1.09-1.50)	1.76 (1.47-2.12)	1.80 (1.45-2.24)	1.97 (1.51-2.57)	
**Pooled results**						
1 kHz						
Model 1	Reference	0.98 (0.78, 1.23)	1.18 (0.89, 1.56)	0.88 (0.60, 1.29)	1.07 (0.68, 1.69)	0.99, 0.0
Model 2	Reference	0.97 (0.77, 1.22)	1.17 (0.88, 1.55)	0.87 (0.59, 1.28)	1.01 (0.64, 1.60)	0.89, 0.0
4 kHz						
Model 1	Reference	1.29 (1.12, 1.48)	1.86 (1.58, 2.19)	1.82 (1.49, 2.23)	2.07 (1.64, 2.62)	0.98, 0.0
Model 2	Reference	1.28 (1.11, 1.48)	1.80 (1.53, 2.13)	1.79 (1.46, 2.20)	1.99 (1.57, 2.52)	0.89, 0.0

Model 1 adjusted for age and sex

Model 2 adjusted for age, sex, BMI, alcohol consumption, leisure-time physical activity, hypertension, diabetes, and occupational noise exposure

[a] For the exclusive HTP user group

Supplementary file Table 5 Dose-response relationship between tobacco product use and bilateral hearing loss in Japanese workers, 2018-2020 (Study II, N=25926)

	Never smoker	Number of cigarettes / HTPs used per day			P for trend*
		1-10	11-20	≥21	
**Exclusive cigarette use**					
1 kHz					
No. of cases / No. of people	184/15987	13/1372	47/2868	7/246	
Odds ratio (95% CI)					
Model 1	Reference	0.83 (0.47, 1.47)	1.35 (0.96, 1.90)	2.15 (0.99, 4.71)	0.04
Model 2	Reference	0.84 (0.47, 1.49)	1.37 (0.97, 1.93)	2.14 (0.97, 4.72)	0.04
4 kHz					
No. of cases / No. of people	330/15987	47/1372	158/2868	22/246	
Odds ratio (95% CI)					
Model 1	Reference	1.38 (1.00, 1.90)	1.92 (1.56, 2.35)	2.84 (1.78, 4.56)	<0.001
Model 2	Reference	1.35 (0.98, 1.87)	1.88 (1.53, 2.32)	2.67 (1.66, 4.30)	<0.001
**Exclusive HTP use**					

1 kHz					
No. of cases / No. of people	184/15987	9/1164	19/2385	1/141	
Odds ratio (95% CI)					
Model 1	Reference	1.00 (0.50, 1.97)	0.82 (0.50, 1.34)	0.67 (0.09, 4.84)	0.40
Model 2	Reference	1.01 (0.51, 2.01)	0.84 (0.51, 1.37)	0.68 (0.09, 4.92)	0.46
4 kHz					
No. of cases / No. of people	330/15987	35/1164	87/2385	12/141	
Odds ratio (95% CI)					
Model 1	Reference	2.01 (1.39, 2.90)	1.68 (1.30, 2.16)	3.83 (2.06, 7.14)	<0.001
Model 2	Reference	2.00 (1.38, 2.89)	1.68 (1.30, 2.17)	3.76 (2.01, 7.06)	<0.001
**Dual use**					
1 kHz					
No. of cases / No. of people	184/15987	3/461	10/1201	1/101	
Odds ratio (95% CI)					
Model 1	Reference	0.81 (0.26, 2.58)	0.82 (0.43, 1.57)	0.79 (0.11, 5.72)	0.49
Model 2	Reference	0.83 (0.26, 2.62)	0.83 (0.43, 1.59)	0.80 (0.11, 5.84)	0.52

4 kHz					
No. of cases / No. of people	330/15987	10/461	61/1201	6/101	
Odds ratio (95% CI)					
Model 1	Reference	1.30 (0.68, 2.50)	2.32 (1.73, 3.10)	1.95 (0.82, 4.64)	<0.001
Model 2	Reference	1.27 (0.66, 2.44)	2.28 (1.70, 3.07)	1.91 (0.80, 4.55)	<0.001

Model 1 adjusted for age and sex

Model 2 adjusted for age, sex, BMI, alcohol consumption, leisure-time physical activity, hypertension, diabetes, and occupational noise exposure

*The P for trend was determined by modeling smoking intensity as a continuous variable, incorporating the median values of each group into the analysis.

©2024 Hu H. et al.
